# Settings, characteristics, and experiences of stigma among people with tuberculosis in Kenya: National survey results

**DOI:** 10.1371/journal.pgph.0005283

**Published:** 2026-06-15

**Authors:** Aiban Ronoh, Joshua Limo, Lazarus Odeny, Stephen Macharia, Rachael Muinde, Joyce Kiarie, Jane Ong’ang’o, Nkirote Mwirigi, Drusilla Nyaboke, Evaline Kibuchi, Timothy Kilonzo, Rose Wandia, L. Nkirote Mugambi-Nyaboga, Immaculate Kathure

**Affiliations:** 1 National Tuberculosis Program, Nairobi, Kenya; 2 Population Services, Nairobi, Kenya; 3 AMREF Health Africa, Nairobi, Kenya; 4 Centre for Health Solutions, Nairobi, Kenya; 5 Centre for Respiratory Diseases Research, KEMRI, Nairobi, Kenya; 6 Stop TB, Nairobi, Kenya; University of Sydney, AUSTRALIA

## Abstract

Tuberculosis (TB) related stigma remains a significant barrier to TB care and treatment adherence in Kenya. Despite progress in TB control, stigma continues to affect individuals diagnosed with TB and their families, leading to delayed healthcare-seeking behaviors, social exclusion, and economic consequences. This study examines the prevalence and dimensions of TB-related stigma among people infected and affected by TB in Kenya. A cross-sectional study was conducted in November 2023. Data were collected from people with TB (PWTB) in 180 health facilities across 11 counties in Kenya. Recruitment started on 11/05/2023 and ended on 11/05/2024. A multistage stratified sampling method was employed to ensure regional representation. Stigma levels were assessed using a structured stigma index, and logistic outputs was used to analyze factors associated with stigma. A total of 357 PWTB were included in the analysis. Most (67%) were men and the median age was 35 years. The study found high levels of community stigma (68%), family stigma (52%), healthcare system stigma (51%) and self-stigma (49%). Further, multivariable logistic regression indicated that participants with secondary or higher education had significantly higher odds of reporting stigma compared with those with primary education or less (Adjusted OR = 9.30, 95% CI: 2.01–57.3, p = 0.008), while female participants had significantly higher odds of reporting stigma compared with males (Adjusted OR = 4.02, 95% CI: 1.14–15.6, p = 0.035). TB-related stigma in Kenya is prevalent across multiple dimensions, with community, family level, healthcare system and self-stigma particularly prominent. Female sex and higher educational attainment were independently associated with reporting stigma. These findings highlight the need for targeted community-based stigma reduction strategies, integration of psychosocial support within TB care, and gender-responsive strategies.

## 1. Introduction

Kenya is among the 30 high TB-burden countries that contribute to 80% of the global burden of TB disease [1] WHO estimates that 124,000 people fell ill due to TB in Kenya in 2023 but only 97,126 TB cases were diagnosed and started on treatment. This represented a case detection rate of 77% with 23% of TB cases being missed [1]. The overall case notification rate for the year 2023 was 204 TB cases per 100,000 population [2], well below the estimated case notification rate established during the prevalence survey conducted in 2016 [3,4]. This is further indication that there are PWTB missed within communities and the health system, highlighting persistent gaps along the TB care cascade, including delays in diagnosis, linkage to treatment and retention in care.

TB control efforts have increasingly become patient-centered, emphasizing universal access to care. A major milestone was the 2018 UN General Assembly high-level meeting on TB, where a political declaration was endorsed to accelerate progress toward ending TB by 2035. Among its key objectives was the elimination of stigma and discrimination, recognizing that addressing stigma is central to creating a supportive environment for PWTB and improving overall TB control [4]. This is particularly important in high-burden settings where social and structural barriers intersect with health system constraints.

Stigma, described as a process of devaluation, involves being discredited, viewed as shameful, or perceived as threatening [5]. Globally, stigma is a well-documented barrier to health-seeking, engagement in care, and treatment adherence [6,7]. Misconceptions about TB transmission and its association with HIV/AIDS fuel social isolation and discrimination. Families may isolate members with TB, particularly women, due to fear of infection and social consequences [8]. Negative attitudes from healthcare providers and operational barriers within health systems further exacerbate stigma, discouraging timely care-seeking [9], thereby reinforcing diagnostic delays and undermining treatment continuity.

Stigma within community settings has been widely documented, where fear of infection and misinformation drive gossip, social isolation, and rejection [10,11]. In Kenya, delayed TB care has been linked to stigma and gender norms, particularly among women and rural dwellers, who face heightened discrimination within families and communities [8,12]. Similarly, in Ghana, stigma has been shown to contribute to the concealment of symptoms and delays in diagnosis, particularly among women [13]. Beyond individual experiences, community-level stigma functions as a structural barrier within the TB response, influencing disclosure, care-seeking trajectories, treatment initiation, and ultimately shaping patterns of ongoing transmission.

Key determinants of TB stigma include socioeconomic factors such as low education, poverty, and lack of access to TB. Patients with low- or no-income report higher internalized stigma, including shame and exclusion, which undermines treatment adherence [14,15]. However, evidence is mixed: a Ugandan study of over 33,000 participants found higher stigma levels among older adults, urban residents, those with higher education, and those with greater TB knowledge, suggesting that awareness alone may not mitigate stigma and can sometimes reinforce it through heightened associations [16]. Religious beliefs and faith-based organizations (FBOs), meanwhile, have demonstrated potential in reducing stigma through psychosocial support and community education [17,18], illustrating the complex interplay between social norms, cultural institutions, and health behaviors within different contexts.

Stigma intensity also varies by treatment phase and drug resistance status. Newly diagnosed patients and those with drug-resistant TB (DR-TB) often report greater stigma due to prolonged treatment, visible side effects, and fear of infecting others [19,20] -. Anticipated stigma, that is, fear of rejection if status is disclosed, has been associated with delayed diagnosis, missed appointments, and poor psychological health [21], with implications for adherence, mental well-being, and long-term treatment outcomes.

Within health facilities, discriminatory practices have also been reported, including negative attitudes by health workers who may blame patients for late presentation or visibly segregate them. Such actions reinforce stigma despite TB being curable [9]. Understanding the origins of TB stigma is therefore integral to reducing its impact on care-seeking behaviors, which often lead to delayed diagnosis, complications, and TB-related mortality [8]. Evidence from Uganda highlights high levels of TB-related stigma with notable geographic disparities, driven by community perceptions, misconceptions, and knowledge gaps [11]. Similarly, in West Pokot, Kenya, stigma was strongly associated with HIV/AIDS and fear of transmission, leading to both perceived and enacted stigma [16].

Overall, TB stigma represents a powerful social label that alters self-perception and social standing. It delays care-seeking, prolongs infectiousness, worsens health outcomes, and drives self-isolation and premature treatment discontinuation [8,9,22]. Misconceptions about the causes of TB and its transmission further exacerbate isolation and discrimination [23]

All in all, the literature demonstrates that TB stigma is multidimensional, rooted in social, economic, cultural, and institutional factors. To date, however, there has been no nationally representative survey of TB-related stigma in Kenya, limiting the ability to quantify its distribution across settings and to design data-driven stigma-reduction interventions at scale. The objective of this study was to estimate the prevalence and dimensions of TB-related stigma among PWTB in Kenya, and to identify factors independently associated with reported stigma.

## 2. Methodology

### Study design

This was a cross-sectional national survey that assessed the prevalence and dimensions of stigma among PWTB within households, communities, and healthcare settings, and was conducted between May 2023 and May 2024.

### Study setting

Kenya is administratively divided into 47 counties grouped into 12 regions for planning and coordination purposes. These counties vary in population size, TB burden, and health system capacity. While some counties, such as Nairobi, Mombasa, and Kisumu, report high TB notification rates and are considered high-burden areas, others have relatively low TB case numbers. HIV prevalence, a key driver of TB, also varies widely across counties—from under 2% in some regions to over 15% in others. Kenya’s health system is decentralized and structured across six levels of care: community health services (Level 1), primary healthcare facilities (Levels 2–3), county referral hospitals (Level 4), and national referral hospitals (Levels 5–6). TB diagnostic services are available at multiple levels, with smear microscopy, Xpert MTB/RIF, Trunat, and digital chest X-rays widely distributed. Advanced diagnostics, including TB culture and drug susceptibility testing (DST), are available at designated regional and national reference laboratories.

### Study population

The target population comprised PWTB who were active on treatment and were 15 years and above.

### Conceptual framework

**Fig 1:**
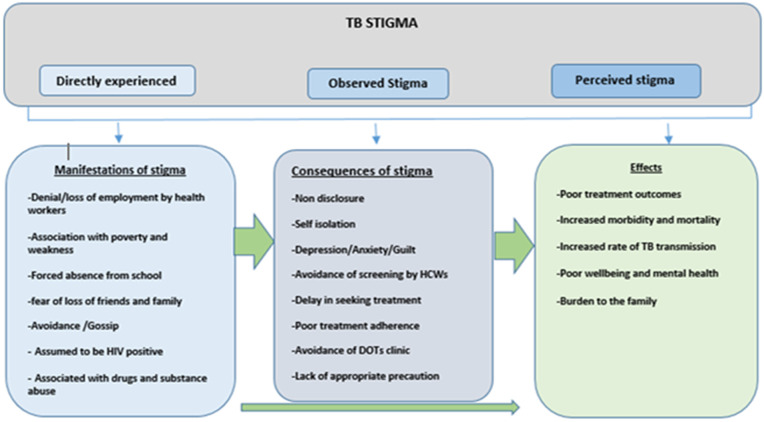
TB stigma index survey conceptual framework (Adapted from Turan & Nyblade) [18].

Fig 1 below shows the conceptual framework that was adapted during the TB stigma index survey in Kenya. It outlines the effects of stigma as perceived by affected persons within the dimensions of health seeking and social perceptions, TB prevention, TB adherence and care, and subsequent TB treatment outcomes. This framework further justifies variable selection and the domains of stigma that were studied.

### Sampling procedures

A multistage stratified sampling method was employed to ensure regional representation across Kenya. The country was stratified into 12 regions: Nairobi North, Nairobi South, Central, Coast, Eastern North, Eastern South, Northeastern, Rift Valley North, Rift Valley South, Nyanza North, Nyanza South, and Western. From each region, one county was purposely selected based on operational feasibility and TB burden. Within each selected county, two sub-counties were further purposely chosen. Criteria for sub-county selection included TB case notification rates (CNR), urban–rural setting, availability of private health facilities providing TB care, and presence of prison health facilities

Within these sub-counties, a total of 180 health facilities offering TB treatment services were identified as primary recruitment sites for participants undergoing TB treatment.

The sample size was calculated using the formula for single population proportion:


$$\begin{array}{c} n\;=\;Z^{\text{2}}p(\text{1}-p)\;/\;d^{\text{2}} \end{array}$$

Where Z = 1.96 at 95% confidence, p = estimated stigma prevalence (assumed 50% due to lack of national estimate), d = margin of error (5%). After adjusting for 10% non-response, the final sample size was 421. Participants were then selected consecutively from TB treatment registers from the selected facilities until the required sample size was reached.

### Eligibility criteria

#### Inclusion criteria.

All PWTB aged 15 years and above, who gave informed consent, were included in the study.

#### Exclusion criteria.

All people who were below the age of 15 years were excluded. Individuals under the influence of alcohol or any substance likely to impair logical reasoning at the time of interview in addition to those who declined to give consent in the study.

### Data collection tools and procedures

Data were collected using a digital questionnaire developed on Open Data Kit (ODK). Trained data collectors administered the tool to eligible participants who provided informed consent. The completed forms were uploaded to a secure web-based system, from which data were extracted, cleaned, and exported to Excel for preliminary review.

Stigma was assessed using a set of standardized questions rated on a **five-point Likert scale** (0 = Strongly Disagree to 4 = Strongly Agree). A total stigma score was computed for each participant by summing individual item scores. These scores were further transformed into a binary variable: participants with scores less than or equal to the neutral midpoint (“Disagree” × number of items) were categorized as having **“no stigma,”** while those scoring above this threshold were categorized as experiencing **“stigma.”**

### Data analysis

Exploratory factor analysis (EFA) was conducted to test the internal consistency and construct validity of the stigma scale in the Kenyan context. Cronbach’s alpha was calculated to assess internal reliability, with values ≥0.7 indicating acceptable consistency. The principal components extraction method was used to identify underlying factors, with factor loadings ≥0.4 considered acceptable.

Data were analyzed using R statistical software (version 5.4.0) for data management and reporting. A working dataset was created by sub setting and recoding key variables including: i) Sociodemographic factors: age group, sex, marital status, education, religion, occupation, place of residence, and family size and ii) psychosocial and clinical variables: perceived social support, substance use, treatment phase, patient type, mental illness, comorbidities, TB type, and stigma setting

Stigma-related responses covering domains such as guilt, fear, social avoidance, and disclosure concerns were numerically encoded (1 = Strongly Agree to 5 = Strongly Disagree). Scores were aggregated row-wise per participant to generate a mean stigma score, which was then categorized as follows: 1.00–1.49: Strongly Disagree, 1.50–2.49: Disagree, 2.50–3.49: Neutral, 3.50–4.49: Agree and 4.50–5.00: Strongly Agree.

For binary classification, participants who “Agreed” or “Strongly Agreed” with stigma-related items were categorized as experiencing stigma, while those who “Disagreed” or “Strongly Disagreed” were categorized as not experiencing stigma. Responses with a “Neutral” score were treated as missing in the binary variable.

Descriptive statistics and cross-tabulations were generated, and inferential analysis was performed using Chi-square tests and binary logistic regression. Variables with p-values <0.05 in bivariate analysis were entered into a multivariate logistic regression model to identify independent predictors of TB-related stigma. Outputs are presented in Table 1 and Table 2 of the results section.

**Table 1:** Demographic characteristics of people with TB.

*Characteristic*	*N = 357* ^*1*^
***Age***	
*15-24*	*58 (16.2%)*
*25-34*	*110 (30.8%)*
*35-44*	*92 (25.8%)*
*45-54*	*51 (14.3%)*
*55-64*	*28 (7.8%)*
*65+*	*18 (5.0%)*
***Sex***	
*Female*	*115 (32.2%)*
*Intersex*	*1 (0.3%)*
*Male*	*241 (67.5%)*
***Marital Status***	
*Divorced*	*10 (2.8%)*
*Married*	*179 (50.1%)*
*Not Applicable*	*6 (1.7%)*
*Separated*	*29 (8.1%)*
*Single*	*113 (31.7%)*
*Widowed*	*20 (5.6%)*
***Education Level***	
*No formal education*	*35 (9.8%)*
*Primary level*	*148 (41.5%)*
*Secondary Level*	*133 (37.3%)*
*Tertiary Level*	*41 (11.5%)*
***Religion***	
*Christians*	*304 (85.2%)*
*Muslim*	*49 (13.7%)*
*None*	*2 (0.6%)*
*Pagans*	*2 (0.6%)*
***Occupation***	
*Business*	*71 (19.9%)*
*Farming*	*61 (17.1%)*
*Formal Employment*	*25 (7.0%)*
*Informal Employment*	*75 (21.0%)*
*Pupils / Students*	*19 (5.3%)*
*Unemployed*	*106 (29.7%)*
***Place of Residence***	
*Rural*	*173 (48.5%)*
*Urban*	*184 (51.5%)*
***Stigma***	
*No Opinion*	*135 (37.8%)*
*No Stigma*	*198 (55.5%)*
*Stigma*	*24 (6.7%)*
^*1*^ *n (%); Median (Q1, Q3)*	

**Table 2:** Bivariate analysis of factors associated with TB stigma.

Characteristic	N	Overall N = 222^1^	No Stigma N = 198^1^	Stigma N = 24^1^	p-value^2^
**Age**	222				0.68
15-24		37 (16.7%)	34 (17.2%)	3 (12.5%)	
25-34		64 (28.8%)	54 (27.3%)	10 (41.7%)	
35-44		59 (26.6%)	52 (26.3%)	7 (29.2%)	
45-54		28 (12.6%)	27 (13.6%)	1 (4.2%)	
55-64		21 (9.5%)	19 (9.6%)	2 (8.3%)	
65+		13 (5.9%)	12 (6.1%)	1 (4.2%)	
**Sex**	222				0.20
Female		76 (34.2%)	65 (32.8%)	11 (45.8%)	
Male		146 (65.8%)	133 (67.2%)	13 (54.2%)	
**Marital Status**	222				0.68
Divorced		5 (2.3%)	5 (2.5%)	0 (0.0%)	
Married		115 (51.8%)	101 (51.0%)	14 (58.3%)	
Not Applicable		3 (1.4%)	3 (1.5%)	0 (0.0%)	
Separated		18 (8.1%)	18 (9.1%)	0 (0.0%)	
Single		67 (30.2%)	59 (29.8%)	8 (33.3%)	
Widowed		14 (6.3%)	12 (6.1%)	2 (8.3%)	
**Education Level**	222				0.16
No formal education		23 (10.4%)	23 (11.6%)	0 (0.0%)	
Primary level		96 (43.2%)	86 (43.4%)	10 (41.7%)	
Secondary Level		75 (33.8%)	63 (31.8%)	12 (50.0%)	
Tertiary Level		28 (12.6%)	26 (13.1%)	2 (8.3%)	
**Religion**	222				0.073
Christians		182 (82.0%)	165 (83.3%)	17 (70.8%)	
Muslim		38 (17.1%)	32 (16.2%)	6 (25.0%)	
None		1 (0.5%)	0 (0.0%)	1 (4.2%)	
Pagans		1 (0.5%)	1 (0.5%)	0 (0.0%)	
**Occupation**	222				0.18
Business		50 (22.5%)	45 (22.7%)	5 (20.8%)	
Farming		40 (18.0%)	38 (19.2%)	2 (8.3%)	
Formal Employment		14 (6.3%)	11 (5.6%)	3 (12.5%)	
Informal Employment		47 (21.2%)	38 (19.2%)	9 (37.5%)	
Pupils / Students		13 (5.9%)	12 (6.1%)	1 (4.2%)	
Unemployed		58 (26.1%)	54 (27.3%)	4 (16.7%)	
**Residence**	222				0.31
Rural		114 (51.4%)	104 (52.5%)	10 (41.7%)	
Urban		108 (48.6%)	94 (47.5%)	14 (58.3%)	
**Family Size**	222	4.00 (3.00, 6.00)	4.00 (3.00, 6.00)	5.00 (3.00, 6.50)	0.57
**Social Support**	222				0.42
Moderate		114 (51.4%)	104 (52.5%)	10 (41.7%)	
Poor		90 (40.5%)	77 (38.9%)	13 (54.2%)	
Strong		18 (8.1%)	17 (8.6%)	1 (4.2%)	
**Substance Use**	185				0.80
Alcohol		26 (14.1%)	25 (14.9%)	1 (5.9%)	
Bhang		1 (0.5%)	1 (0.6%)	0 (0.0%)	
Miraa (khat)		4 (2.2%)	4 (2.4%)	0 (0.0%)	
None		149 (80.5%)	133 (79.2%)	16 (94.1%)	
Other (specify)		2 (1.1%)	2 (1.2%)	0 (0.0%)	
Tobacco use		3 (1.6%)	3 (1.8%)	0 (0.0%)	
**Phase**	216				0.091
Continuation phase		134 (62.0%)	124 (63.9%)	10 (45.5%)	
Intensive phase		82 (38.0%)	70 (36.1%)	12 (54.5%)	
**Patient Type**	222				0.58
New		189 (85.1%)	169 (85.4%)	20 (83.3%)	
Relapse		20 (9.0%)	18 (9.1%)	2 (8.3%)	
Retreatment		9 (4.1%)	8 (4.0%)	1 (4.2%)	
Treatment after Loss to Follow Up		3 (1.4%)	2 (1.0%)	1 (4.2%)	
Treatment Failure		1 (0.5%)	1 (0.5%)	0 (0.0%)	
**Mental Illness**	220	19 (8.6%)	18 (9.2%)	1 (4.2%)	0.70
**Comorbidities**	213				0.93
Covid		2 (0.9%)	2 (1.1%)	0 (0.0%)	
Diabetes		6 (2.8%)	6 (3.2%)	0 (0.0%)	
HIV		35 (16.4%)	32 (16.9%)	3 (12.5%)	
None		163 (76.5%)	143 (75.7%)	20 (83.3%)	
Other (specify)		7 (3.3%)	6 (3.2%)	1 (4.2%)	
**Settings of Stigma**	173				<0.001
C1. Hospitals/Clinics		7 (4.0%)	6 (3.7%)	1 (8.3%)	
C2. Community/Neighbours		25 (14.5%)	18 (11.2%)	7 (58.3%)	
C3. Home/Family		4 (2.3%)	4 (2.5%)	0 (0.0%)	
C4. Workplace		10 (5.8%)	8 (5.0%)	2 (16.7%)	
C5. Prison		13 (7.5%)	13 (8.1%)	0 (0.0%)	
C6. Rescue Centres		1 (0.6%)	1 (0.6%)	0 (0.0%)	
C7. Others		1 (0.6%)	1 (0.6%)	0 (0.0%)	
C8. None		112 (64.7%)	110 (68.3%)	2 (16.7%)	
**TB Type**	219				0.36
Don’t Know		34 (15.5%)	29 (14.9%)	5 (20.8%)	
DR-TB		5 (2.3%)	4 (2.1%)	1 (4.2%)	
DS-TB		180 (82.2%)	162 (83.1%)	18 (75.0%)	

^1^n (%); Median (Q1, Q3), ^2^Fisher’s exact test; Pearson’s Chi-squared test; Wilcoxon rank sum test

### Ethics statement

Ethical approval was obtained from **Amref Health Africa Ethics and Scientific Review Committee (ESRC), approval number -AMREF-ESRC P1354|2022** on May 11^th,^ 2023. Research clearance was granted by the National Commission for Science, Technology, and Innovation (NACOSTI/P/23/29356).

All study procedures were conducted in accordance with national ethical guidelines and the principles of the Declaration of Helsinki. Participation was voluntary and eligible participants were provided with detailed information about the study’s purpose, procedures, potential risks and benefits, confidentiality measures, and their rights, including the right to decline or withdraw at any time without consequences to their clinical care. Written informed consent was obtained from all participants prior to enrollment. Only those who signed the consent form were included in the study. Participant confidentiality was maintained through de-identification of data, secure storage of electronic records on password-protected systems, and restricted access to study data limited to authorized research personnel.

## 3. Results

### Participant demographics

Of the 421 eligible participants approached, 367 consented (87% response rate). After excluding incomplete stigma responses (n = 10), 357 were included in descriptive analysis. Of these, 228 provided complete stigma responses used for regression analysis.

A total of 35 7 individuals with TB participated in the descriptive analysis (Table 1), with the largest age group being 25–34 years (30.8%). Most participants were male (67.5%) and resided in urban areas (51.5%), while 48.5% lived in rural settings. In terms of education, 41.5% had completed primary school, and 9.8% reported having no formal education. Half of the participants were married, and the majority (85.2%) identified as Christian. Regarding employment status, 29.7% were unemployed.

### Tuberculosis-related stigma dimensions

**Fig 2:**
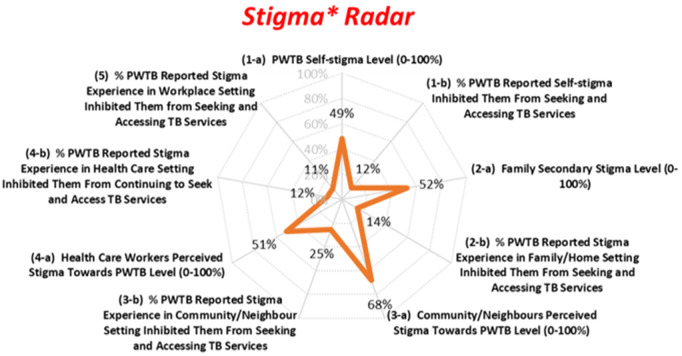
Stigma Radar* adapted from the Stop TB Partnership stigma assessment handbook [24].

The stigma radar was used to assess the level of stigma among study respondents across five dimensions: self-stigma (patient experience), and secondary stigma from family, community, healthcare settings, and workplaces. As shown in Fig 2, 49% of PWTB reported experiencing self-stigma, with 12% stating it had prevented them from seeking or accessing TB services. Secondary stigma within the family was reported by 52% of participants; however, only 14% indicated it posed a barrier to care.

Community-level stigma was most prevalent, with 68% of respondents reporting stigma from neighbors or the broader community, and 25% stating that it negatively impacted their access to TB services. Perceived stigma within healthcare settings was reported by 51% of participants, with 12% identifying it as a barrier to care. Workplace-related stigma was noted by 11% of respondents as a deterrent to seeking TB services.

### Factors associated with TB stigma

Table 2 below summarizes the social demographic, and clinical factors associated with TB stigma. Out of 357 participants, 228 individuals with TB shared their experiences regarding stigma. Among them, 24 (11%) reported experiencing TB-related stigma, while 204 (89%) did not. The remaining 139 participants did not provide an opinion and were excluded from the bivariate analysis.

Most demographic and clinical characteristics—including age, sex, marital status, education, occupation, residence, substance use, treatment phase, mental illness, and comorbidities, were not statistically associated with reported stigma (all *p* > 0.05). However, the setting in which stigma was experienced showed a significant association (*p* < 0.001). Among those who reported stigma, 58% identified the community or neighborhood as the primary source, making it the most prevalent setting in which stigma was experienced. Stigma in healthcare settings (8.3%) and workplaces (17%) was less commonly reported, and no respondents reported experiencing stigma at home, in prisons, or in rescue centers.

While not statistically significant, stigma was most frequently reported among individuals aged 25–44 years (71%), males (54%), and those with secondary-level education (50%). Informal workers and business persons represented the highest proportions of employed individuals reporting stigma. Additionally, those in the intensive phase of TB treatment reported more stigma (55%) than those in the continuation phase (45%), though this difference did not reach statistical significance (*p* = 0.089).

### Logistic modelling

In the multivariable logistic regression model (Table 3), educational attainment and sex emerged as independent predictors of TB-related stigma. Participants with secondary or higher education had significantly higher odds of reporting stigma compared with those with primary education or less (Adjusted OR = 9.30, 95% CI: 2.01–57.3, p = 0.008). Female/other participants also had significantly higher odds of reporting stigma compared with males (Adjusted OR = 4.02, 95% CI: 1.14–15.6, p = 0.035).

**Table 3:** Factors associated with TB stigma (crude and adjusted ORs).

	Crude OR (95% CI)				Adjusted OR (95% CI)		
Characteristic	N	OR	95% CI	p-value	OR	95% CI	p-value
**Age**	222						
15-34		—	—		—	—	
35-54		0.69	0.26, 1.71	0.4	1.12	0.20, 6.16	0.9
55+		0.66	0.14, 2.20	0.5	3.81	0.50, 29.7	0.2
**Sex**	222						
Male		—	—		—	—	
Female/Other		1.73	0.72, 4.08	0.2	4.02	1.14, 15.6	0.035
**Marital Status**	219						
Married / Partnered		—	—		—	—	
Single / Never married		0.98	0.37, 2.42	>0.9	1.20	0.26, 5.34	0.8
Previously married		0.41	0.06, 1.57	0.3	0.50	0.05, 3.26	0.5
**Education Level**	222						
Primary or less		—	—		—	—	
Secondary or higher		1.71	0.73, 4.15	0.2	9.30	2.01, 57.3	0.008
**Residence**	222						
Urban		—	—		—	—	
Rural		0.65	0.27, 1.51	0.3	0.98	0.28, 3.37	>0.9
**Social Support**	222						
Moderate		—	—		—	—	
Poor		1.76	0.73, 4.31	0.2	1.65	0.40, 6.96	0.5
Strong		0.61	0.03, 3.51	0.6	0.47	0.02, 4.27	0.6
**Substance Use**	185						
No		—	—		—	—	
Yes		0.24	0.01, 1.23	0.2	0.41	0.02, 2.60	0.4
**Phase**	216						
Continuation phase		—	—		—	—	
Intensive phase		2.13	0.87, 5.28	0.10	1.91	0.59, 6.29	0.3
**Patient Type**	222						
New		—	—		—	—	
Previously treated		1.17	0.32, 3.36	0.8	0.69	0.03, 4.57	0.7
**Comorbidities**	213						
No		—	—		—	—	
Yes		0.62	0.17, 1.75	0.4	0.94	0.16, 4.40	>0.9

Abbreviations: CI = Confidence Interval, OR = Odds Ratio

No other demographic or clinical characteristics, including age, marital status, place of residence, perceived social support, substance use, phase of treatment, patient type, or comorbidity—were significantly associated with TB-related stigma after adjustment.

Overall, these findings indicate that educational attainment and sex were the key independent predictors of TB-related stigma in this adult population, while other demographic and clinical factors did not independently influence stigma levels.

The multivariable logistic regression model demonstrated adequate fit to the data. Model calibration was assessed using the Hosmer–Lemeshow goodness-of-fit test with five groups (χ² = 5.64, df = 3, p = 0.13), indicating no evidence of lack of fit. The model demonstrated good discrimination between participants with and without TB-related stigma (an area under the receiver operating characteristic curve (AUC) = 0.82, 95% CI: 0.72–0.92).

## 4. Discussion

This national cross-sectional study provides the first multi-county assessment of TB-related stigma among PWTB in Kenya. Among the 35 7 surveyed, nearly half reported experiencing some form of stigma, with community-level stigma being the most prevalent. However, only 11% were classified as experiencing significant stigma based on the binary composite score, suggesting that while stigma is widespread in perception, the intensity and actionable burden may vary across individuals and contexts.

Although stigma reporting varied by age, sex, education, and residence, none of these were statistically significant predictors. This contrasts with findings from other contexts. In Uganda, higher education was paradoxically linked to greater stigma, possibly due to heightened awareness of TB/HIV associations [16]. Conversely, in Colombia, low education and homelessness were associated with increased stigma [25]. Informal employment was the most common occupation among those reporting stigma (38%), followed by business owners (21%). Although not statistically significant, this trend is consistent with evidence from Uganda and Colombia, where unemployment and homelessness heightened vulnerability to stigma [16,25].

Stigma was most pronounced in community settings (68%), followed by family (52%), healthcare settings (51%), and internalized stigma (49%). These findings mirror previous studies from Kenya, South Africa and Uganda, where gossip, visible symptoms, fear of infection, misconceptions regarding TB transmission, and its perceived association with HIV drive social distancing and concealment behaviors [8,26–29]. Although healthcare-related stigma was also reported, its prevalence was lower compared to community-level stigma, suggesting that interventions targeting community awareness may yield substantial benefit.

In multivariable analysis, female sex and higher educational attainment were independently associated with reporting stigma. Other demographic and clinical factors were not statistically significant predictors. Of note, women had four times higher odds of reporting stigma compared to men. This finding is consistent with literature suggesting that women may face compounded stigma related to gender norms, caregiving roles, and fears of social exclusion [12]. Interestingly, participants with secondary education or higher had significantly greater odds of reporting stigma. Similar findings have been reported in Uganda [16], where increased awareness may heighten perceived stigma or fear of social labeling. However, the wide confidence intervals observed in our study suggest cautious interpretation and warrant further exploration.

Other demographic and clinical factors including age, residence, treatment phase, social support, and comorbidities were not independently associated with stigma. Although stigma appeared more frequently reported during the intensive phase of treatment, this did not reach statistical significance. These findings underscore that stigma in this setting may be less driven by clinical characteristics and more by sociocultural context.

This study has several limitations. First, participants were recruited from health facilities and therefore represent individuals already engaged in care, potentially underestimating stigma among those who avoid services. Second, exclusion of neutral responses in binary classification reduced the analytical sample and may affect generalizability. Third, stigma was self-reported and subject to social desirability bias. Finally, the cross-sectional design precludes causal inference.

Despite these limitations, this study provides important national-level insights into TB-related stigma in Kenya. Interventions should prioritize community engagement strategies, gender-sensitive approaches, and integration of psychosocial support within TB care programs. Addressing stigma at multiple levels remains critical to improving timely care-seeking, treatment adherence, and progress toward ending TB in Kenya.

## Supporting information

S1 Data Stigma data. (CSV)
