# Supplementary material for: Settings, characteristics, and experiences of stigma among people with tuberculosis in Kenya: National survey results
Source: PLOS Glob Public Health. 2026 Jun 15;6(6):e0005283. doi: 10.1371/journal.pgph.0005283 (PMC13268150; doi:10.1371/journal.pgph.0005283)
Supplement: Attachment — Submitted filename: Response Tracker_Final version.docx [file pgph.0005283.s005.docx]

**Manuscript Reviewers Comments and Response**

| **Page** | **Section** | **Line** | **Issue** | **Response** |
| --- | --- | --- | --- | --- |
| 1 | Abstract | 32 | Self-stigma results need to be suggested in the conclusion | Done |
|  |  | 43-44 | missing post stop after the first sentence line 43  Line 43 and line 44 please reconsider the use of TB versus tuberculosis. Also review the entire background and document and consider of using TB once the acronym has been established | Done |
| 2 | Introduction |  | In the background, please ensure that you differentiate between literature or studies that were conducted among TB patients and also those that were conducted among the community members, since your study is among TB patients.  The paragraphs seem a bit too short and lack an overarching structure to guide the review. |  |
|  |  | 58 | (6) (7) (8) Put in the right reference format (6-8) | Done |
|  |  | 60-61 | Mbuthia et al, 2020 (Put in the right reference format- Vancouver) | Done |
|  |  | 64 | (10) (11) Put in the right reference format (10-11) | Done |
|  |  | 66 | (12) (13) (14) Put in the right reference format (12-14) | Done |
|  |  | 71 | (16) (17) Put in the right reference format (16-17) | Done |
|  |  | 77 | (19) (20) Put in the right reference format (19-20) | Done |
|  |  | 79 | Align (DR-TB) how is written in the paper | Done |
|  |  | 80 | (21) (22) (23) Put in the right reference format (21-23) | Done |
| 3 |  | 94 | (12) (24) (9) Put in the right reference format | Done |
|  |  | 98-100 | *“This study, therefore sought to fill that gap by assessing the levels and dimensions of stigma experienced by people with TB, generating evidence to inform targeted interventions and contribute to the broader goal of ending TB in Kenya.”* – **Is this an objective? Needs to be checked**. | Done |
|  |  | 101 | I suggest the conceptual framework be put in the Methods section. | Done |
|  | Methodology |  | The methods seem not appropriately organized according to PLOS’s typical requirements; for instance, the Ethical Statement seems to come first instead. Moreover, it seems incomplete; it is not just about the approvals but also about how the study procedures adhered to known stands.  Include the model-building process for the logistic regression and other considerations made for a parsimonious model. | Done (reorganized) |
| 4 |  | 135 | 15 years (There were some participants aged under 15 years) | Addressed |
|  |  | 139 | 12 regions? - Confirm | Addressed |
| 5 |  | 147 | 421- show the formula for sample size calculation & Reference | Addressed |
|  |  | 153 | Participants aged <18 years need to have informed consent from parents | Addressed |
|  |  | 160 | Details of questionnaire & reference | Addressed |
|  |  | 164 | Relate with data analysis on page 6 | Addressed |
|  |  | 167 | Relate with data analysis | Addressed |
|  |  | 168 | Relate with data analysis | Addressed |
|  |  | 169 | Relate with data analysis | Addressed |
|  |  | 173 | >0.7, show exact value | Addressed |
|  |  | 181 | TB type, the author used ‘treatment type’ so need to e same | Addressed |
| 6 |  | 189 | How many items did you use to categorise into binary classification? | Addressed |
|  |  | 192 & 193 | These statistics were not used and shown in the results | Addressed |
|  | Results |  | Persons with TB, people with TB, PWTB, - It would be good to harmonize all these. Moreover, it would be good to establish the abbreviation, and use it consistently. Similar my comment regarding TB and Tuberculosis.  The inclusion and exclusion criteria mentions that only PWTB 15 years and older were interviewed, however, the results show that those who were 0 – 4 years were 10 PWTB. How do you reconcile this?  This study mentions that “multivariate logistic regression was used to analyze factors associated with stigma” (see abstract and line 192 ) but the results that support this are missing. It looks like the analysis stopped at cross-tabulation or bivariate analysis. | Done |
|  |  | 202 | Line 202 – Explain why this is less than the sample calculated first. |  |
| 6-7 | Table 1 | 208 | Check exclusion criteria  Use two decimal points | Done |
| 7 |  | 213 | Use obstructed in place of prevented | Done |
| 8 |  | 226 | TB stigma- Check about how to categorize into binary | Addressed |
|  |  | 227 | Line 227 – 228 – What does it mean “Out of 367 participants, 228 individuals with TB shared their experiences regarding stigma” – Did the others decline? Were they not asked their experience, how does this affect the overall sample’s validity? | Addressed |
| 9 | Table 2 | 9-10 | Check exclusion criteria  Use two decimal points  Align (DR-TB) how is written in the paper | Done |
|  | Discussion |  | Overall, I think the discussion is weak. First, the subtitles are not needed in this section. Second, it needs to stick to the significant findings of the study. Third, for instance, line 279 can’t be that community settings emerged as the “leading source”… compared to what sources that were also contributing? How is it a driver? Logistic regression results are missing. How did religious context influence TB stigma? The results presented are not significant, and even if they were, it would be just descriptive analysis. Stick to what matters, regarding the demographics, what matters is how stigma is distributed within the demographics, not how TB is. This is a TB stigma study. You should limit making conclusions that don’t support your analysis. | Done fully |
| 10 |  | 248 | How about the participants | Addressed |
| 11 |  | 259 | 80% confirm | Addressed |
|  |  | 259 | Where is 14% from? | Addressed |
|  |  | 268 | (18) (28) Put in the right reference format | Done |
|  |  | 273 | (12) (29) (30) Put in the right reference format | Done |
|  |  | 281 | (29) (31) (32) Put in the right reference format | Done |
|  |  | 285 | (34) (35) Put in the right reference format | Done |
| 12 |  | 290 | (12) (37) (38) Put in the right reference format | Done |
|  |  | 293 | (36) (20) Put in the right reference format | Done |
|  | Reference | 313 | Put in the right reference format- Vancouver | Done |
|  |  |  |  |  |
|  |  |  |  |  |
|  |  |  |  |  |
