# Supplementary material for: Settings, characteristics, and experiences of stigma among people with tuberculosis in Kenya: National survey results
Source: PLOS Glob Public Health. 2026 Jun 15;6(6):e0005283. doi: 10.1371/journal.pgph.0005283 (PMC13268150; doi:10.1371/journal.pgph.0005283)
Supplement: Attachment — Submitted filename: Tracker_Response to Reviewers Comments -21_04_2026.docx [file pgph.0005283.s006.docx]

| Page | Reviewers Comment | Response |
| --- | --- | --- |
| Page 4 | Methods claim 12 regions, but the list contains 11 named regions (Nairobi North and Nairobi South, Central, Coast, Eastern North, Eastern South, Northeastern, Rift Valley North, Rift Valley South, Nyanza North, Nyanza South, Western). | The missing region added |
| Page 5 | Methods mention Cronbach’s alpha threshold (≥0.7) but no reported alpha value is provided. | Resolved |
| Page 6 | Do we have the dates of ethical approval and NACOSTI. Maybe these could be included. | Date added |
| Page 7 | “Pagans”. Replace with a neutral category label (e.g., “Traditional/Indigenous faith”, “Other religion”, or local program-accepted terminology). | Resolved |
|  | Body text uses “DR‑TB” (with hyphen), but Table 2 uses “DRTB” and “DSTB” (without definitions in text) | Resolved |

**Reviewers Comments & Response Tracker:**
