# Supplementary material for: Settings, characteristics, and experiences of stigma among people with tuberculosis in Kenya: National survey results
Source: PLOS Glob Public Health. 2026 Jun 15;6(6):e0005283. doi: 10.1371/journal.pgph.0005283 (PMC13268150; doi:10.1371/journal.pgph.0005283)
Supplement: Peer review history

# Peer review: Decision Letter 0

**DOI:** 10.1371/journal.pgph.0005283.r001

**Authors:** Graeme Hoddinott

5 Jan 2026

PGPH-D-25-02705

Settings, Characteristics, and Experiences of Stigma Among People with Tuberculosis in Kenya: National Survey Results

PLOS Global Public Health

Dear Dr. Ronoh,

Thank you for submitting your manuscript to PLOS Global Public Health. After careful consideration, we feel that it has merit but does not fully meet PLOS Global Public Health’s publication criteria as it currently stands. Therefore, we invite you to submit a revised version of the manuscript that addresses the points raised during the review process.

==============================

EDITOR:

The reviewers agree that your manuscript addresses an important topic. They have also raised a number of well-justified concerns and points requiring clarification. I hope that you see these as opportunities to further improve your manuscript such that it may be accepted for publication.

==============================

Please submit your revised manuscript by 26 Jan 2025. If you will need more time than this to complete your revisions, please reply to this message or contact the journal office at globalpubhealth@plos.org. When you're ready to submit your revision, log on to https://www.editorialmanager.com/pgph/ and select the 'Submissions Needing Revision' folder to locate your manuscript file.

Please include the following items when submitting your revised manuscript:

- A letter that responds to each point raised by the editor and reviewer(s). You should upload this letter as a separate file labeled 'Response to Reviewers'.
- A marked-up copy of your manuscript that highlights changes made to the original version. You should upload this as a separate file labeled 'Revised Manuscript with Track Changes'.
- An unmarked version of your revised paper without tracked changes. You should upload this as a separate file labeled 'Manuscript'.

Guidelines for resubmitting your figure files are available below the reviewer comments at the end of this letter.

We look forward to receiving your revised manuscript.

Kind regards,

Graeme Hoddinott, Ph.D

Academic Editor

PLOS Global Public Health

Journal Requirements:

1. Please provide a complete Data Availability Statement in the submission form, ensuring you include all necessary access information or a reason for why you are unable to make your data freely accessible. If your research concerns only data provided within your submission, please write "All data are in the manuscript and/or supporting information files" as your Data Availability Statement.

2. Thank you for including an Ethics Statement for your study. Please include (for human participants/donors) - A statement that formal consent was obtained (must state whether verbal/written) OR the reason consent was not obtained (e.g. anonymity). NOTE: If child participants, the statement must declare that formal consent was obtained from the parent/guardian.

3. Please provide separate figure files in .tif or .eps format.

For more information about figure files please see our guidelines:

https://journals.plos.org/globalpublichealth/s/figures

https://journals.plos.org/globalpublichealth/s/figures#loc-file-requirements

If the reviewer comments include a recommendation to cite specific previously published works, please review and evaluate these publications to determine whether they are relevant and should be cited. There is no requirement to cite these works unless the editor has indicated otherwise.

[Note: HTML markup is below. Please do not edit.]

Reviewers' comments:

Reviewer's Responses to Questions

**Comments to the Author**

1. Does this manuscript meet PLOS Global Public Health’s publication criteria? Is the manuscript technically sound, and do the data support the conclusions? The manuscript must describe methodologically and ethically rigorous research with conclusions that are appropriately drawn based on the data presented.

Reviewer #1: Partly

Reviewer #2: No

Reviewer #3: Partly

**********

2. Has the statistical analysis been performed appropriately and rigorously?

Reviewer #1: No

Reviewer #2: No

Reviewer #3: Yes

**********

3. Have the authors made all data underlying the findings in their manuscript fully available (please refer to the Data Availability Statement at the start of the manuscript PDF file)?

The PLOS Data policy requires authors to make all data underlying the findings described in their manuscript fully available without restriction, with rare exception. The data should be provided as part of the manuscript or its supporting information, or deposited to a public repository. For example, in addition to summary statistics, the data points behind means, medians and variance measures should be available. If there are restrictions on publicly sharing data—e.g. participant privacy or use of data from a third party—those must be specified.

Reviewer #1: Yes

Reviewer #2: No

Reviewer #3: No

**********

4. Is the manuscript presented in an intelligible fashion and written in standard English?

PLOS Global Public Health does not copyedit accepted manuscripts, so the language in submitted articles must be clear, correct, and unambiguous. Any typographical or grammatical errors should be corrected at revision, so please note any specific errors here.

Reviewer #1: Yes

Reviewer #2: No

Reviewer #3: Yes

**********

5. Review Comments to the Author

Please use the space provided to explain your answers to the questions above. You may also include additional comments for the author, including concerns about dual publication, research ethics, or publication ethics. (Please upload your review as an attachment if it exceeds 20,000 characters)

Reviewer #1: The author wrote this manuscript quite well. However, there are some suggestions for improving it better including,

Abstract:

The abstract is written well. However, the results showed about self-stigma representing at 49% so this result should be suggested in conclusion as well.

Introduction:

The introduction is organised and written well. However, there are some suggestions about referencing that should be revised along with Vancouver style and the journal format, such as (6)(7)(8), should be (6-8) or (Mbuthia et al., 2020) should be a number of reference. Another point, the abbreviation of drug-resistant TB (DR-TB) should be the same with DRTB in table 2 (page 10). Moreover, in terms of objective of the study, it should be written clearly. The author stated in line 98-100 (page 3), but it seems like expected outcomes rather than its objectives.

Methodology:

Ethics statement:

It is a clear statement, however, the date of approval should be presented as well to ensure the data were collected after approval.

Study population:

The author stated that the target population comprised people with TB who were on treatment and were 15 years and above. However, the results showed that there were some participants aged under 15 years (0-14) as well. Thus, the author should revise and make it correct.

Sampling procedures:

The author stated that the data were collected in 12 regions, however there were only 11 regions stated in line 139-140.

Sample size:

The author showed some details of sample size calculation that met 421 persons (line 147). However, there were only 367 participants recruited to this study which is less than the appropriate sample size calculated. So, the author needs to explain more details about the sample size. It should be 421 as the result of calculation with the appropriate formula. Moreover, if there are sources of the number used to calculate, the reference needed to be stated as well.

Eligibility criteria:

In terms of inclusion and exclusion criteria, the author stated that all people with TB aged 15 years and older would be recruited to the research and all people who were below the age of 15 years were excluded. This is the main point that needs to be clarified because in the results, there are some participants aged 0-14 years as stated before. Moreover, in terms of ethics, participants aged less than 18 years cannot sign the consent form by themselves, their parents should sign the consent form. So, the author needs to revise and clarify further.

Data collection tools and procedures:

There is no information about the questionnaire well. The questionnaire should be clarified the details, especially the items used to categorise into "no stigma and stigma (binary). If you use only one item, it should not be appropriate to categorise. This is an important point of this study that needs to be explained. As well as, if the questionnaire was conducted by previous researchers, it should be cited correctly. Moreover, the author stated in this part that stigma was assessed using a set of standardised questions rated on a five-point Likert scale (0 = Strongly disagree to 4 = Strongly agree), while, in page 6, the author used (1 = Strongly agree to 5 = Strongly disagree) as well as the data were categorised in to 5 groups staring from 1.00 - 5.00. Please check the details again.

Data analysis:

The Cronbach's alpha value needs to be presented with the exact value instead of >=0.70 that it will present the reliability of tools better. Moreover, the author stated in line 181 and table 2 "TB type" but in the conclusion, the author used "treatment type". So, this point needs to be the same. For the binary classification, the author needs to explain more details about how to categorise into 2 groups: no stigma and stigma. In terms of inferential statistics, the binary logistic regression and multiple logistic regression were not used and shown in the results. So, the author needs to revise about this point again.

Results:

The sum of percentage, the details in Table 1 & 2 showed the percentage of each variable, which is good. However, the author needs to check the sum of each variable should be 100%. The author may use two decimal points for presenting the percentage. Moreover, some sub-variables which there is no data (0) does not need to present in the table. Please find the details in the attached file.

In table 2, inferential statistics, the author stated in data analysis that binary logistic regression and multiple logistic regression would be used to analyse to identify the predictors. However, in the results, there is no any results based on these statistics. So, the author needs to revise about the statistics stated in data analysis. Moreover, about chi-square, the author needs to check the assumption of chi-square because No cell should have an expected frequency < 1, and at least 80% of cells should have expected frequencies of 5 or more. So, if it does not meet the assumption, its results might be wrong.

Aged 0-14, the author stated in the methodology that the participants need to be 15 years or over. So, please check the data again.

Some words should be changed for example in line 213 from prevented to obstructed. Moreover, about abbreviation "DSTB and DRTB", for DSTB, the author did not state before so it needs to be mentioned in previous part first before using in this part. As well as, DRTB, the author used DR-TB in line 79 so it needs to be the same, with or without -.

Discussion:

The author wrote this part quite well, however, the author needs to check about the number of percentage presented in this part again. Moreover, citation should be revise and rewrite following the format.

References:

In terms of references, the author should check the format of Vancouver style referencing in both in-cited and references part again. As well as, the author needs to check the format along with the journal format. For example, (xx) to [xx]. Please revise and rewrite following the formats.

Reviewer #2: Please see my report. I think the manuscript transition from a dissertation to a paper is incomplete. Please review my report for details. I raise concerns regarding the sampling, statistical analysis and the conclusions made regarding the results.

Reviewer #3: This cross-sectional study tackles an important global health problem, TB-related stigma among people with TB. This study has significant merits including 367 people living with tuberculosis sampled over one year; and captures various contexts, specifically 180 health facilities across 11counties aiming for a nationally representative survey of TB-related stigma in Kenya. Two hundred and twenty-eight patients provided information regarding TB-related stigma, of whom 24 reported experiencing TB-related stigma.

Several areas remain unclear to me and require further clarification, elaboration or consideration for reformatting.

1. The referencing style used is inconsistent. e.g. introduction section lines 60-61(Mbuthia et al. 2020), whereas other areas have a different style that is numbered. Consider reformatting for consistency.

2. Previous research on TB-related stigma measurement and its implications to TB related outcomes in the Kenyan context has not been highlighted.

3. Ethics statement section could be aligned for consistent formatting with other text sections of the manuscript.

4. The sample size calculation could be further clarified for the readers to judge its robustness.

a. Is there a proportion of TB-related stigma assumed from a previous study?

b. What is the rationale of a 90% response rate? – Lines 147 to 150.

c. What was the actual response rate?

5. From the manuscript, the sample size calculated was 421 TB patients, but only 367 are reported and 228 TB patients provide information related to TB stigma. These patients were sampled over one year from 180 health facilities across 11 counties in Kenya.

a. Further clarification on the sampling frame is needed.

b. How many patients were sampled per health facility? Was there any gender consideration per health facility?

c. How were the 12 regions chosen and how do they relate to the current national or programmatic divisions?

d. It is indicated that one county was chosen from the 12 regions but only 11 counties included.

6. Elaborating on the tool and procedures used is needed for the readers to judge the robustness of the methodology used. This information is crucial in the methods section.

Lines 164-165: “Stigma was assessed using a set of standardized questions rated on a five-point Likert scale (0 = Strongly Disagree to 4 = Strongly Agree).”

(i) What is the set of standardized questions?

(ii) What tool was used?

(iii) Has this tool been previously used in the literature?

(iv) Has the tool been previously used in the Kenyan context?

(v) Is this a validated tool?

(vi) In what language/s were the questions asked?

(vii) Who administered the survey?

Provide relevant references.

These details are missing in the methods section; and need to be considered for inclusion in the main text and/or supplementary material based on journal guidelines.

7. What do the authors think could be the implications of handling neutral scores as missing? Lines 189-190: ‘Responses with a "Neutral" score were treated as missing in the binary variable.’ Please elaborate and describe the possible limitation.

8. Lines 192 to 194: “Variables with p-values <0.05 in bivariate analysis were entered into a multivariate logistic regression model to identify independent predictors of TB-related stigma” – Do the authors mean a multivariable logistic regression model?

9. In the results section, 10 participants are aged 0-14 years however, one of the study inclusion criteria is that participants should be aged 15 years and above. Further clarification is needed.

10. Are the age group categories shown in Table 1 meaningful? Would other summary descriptive statistics for age central tendency and dispersion be considered to provide more information about patient characteristics.

11. The term “Pagan” in Table 1 may be considered derogatory – consider an alternative word.

12. Several other participant characteristics would be important to understand TB-related stigma, including: a) the type of tuberculosis; b) the timing of treatment for the TB patient at which this survey was being performed; c) disclosure of a TB diagnosis; among others. There is existing global, regional and particularly Kenyan literature that supports the importance of these particular characteristics. Consider including these in Table 1.

13. Lines 228-230: “Out of 367 participants, 228 individuals with TB shared their experiences regarding stigma. Among them, 24 (11%) reported experiencing TB-related stigma, while 204 (89%) did not. The remaining 139 participants did not provide an opinion and were excluded from the bivariate analysis.”

a. Based on this statement, it is not clear what the procedures for study participation were. The study was to assess TB-related stigma, but 139 participants did not provide an opinion. Please elaborate the study procedures for the readers to gain clarity.

b. What are the characteristics of the individuals of TB patients who did not share their experiences regarding stigma?

c. Were they different from those who did?

14. Clarification is needed regarding the proportions of stigma provided in different sections of the manuscript. TB-related stigma dimensions in Figure 2 report relatively high TB-related stigma levels (49% for self-stigma, 68% of community-level stigma); compared to the overall TB-related stigma reported as 11% and also shown in Table 2.

15. Consider including whether the type of TB was pulmonary or not, in Table 2. This is not clear.

16. Data analysis:

Lines 171-175: “Exploratory factor analysis (EFA) was conducted to test the internal consistency and construct validity of the stigma scale in the Kenyan context. Cronbach’s alpha was calculated to assess internal reliability, with values ≥0.7 indicating acceptable consistency. The principal components extraction method was used to identify underlying factors, with factor loadings ≥0.4 considered acceptable.”

- Although this section is included in the data analysis methods section, there is no data in the manuscript to support this. Please provide this information if it is available.

Lines 182-186: “Stigma-related responses covering domains such as guilt, fear, social avoidance, and disclosure concerns were numerically encoded (1 = Strongly Agree to 5 = Strongly Disagree). Scores were aggregated row-wise per participant to generate a mean stigma score, which was then categorized as follows: 1.00–1.49: Strongly Disagree, 1.50–2.49: Disagree, 2.50–3.49: Neutral, 3.50–4.49: Agree and 4.50–5.00: Strongly Agree.”

- Similarly, although this section is included in the data analysis methods section, there is no data in the manuscript to support this. Stigma is reported as a binary variable and not continuous. Please provide this information if it is available.

Lines 192-194: Variables with p-values <0.05 in bivariate analysis were entered into a multivariate logistic regression model to identify independent predictors of TB-related stigma. Outputs are presented in Table 1 and Table 2 of the Results section.

- Again, this section is included in the data analysis methods section, but there is no data in the manuscript to support this. No results are provided for multivariable logistic regression in Table 1 or Table 2. Please provide this information if it is available.

Was there a justification of including age group instead of age as a continuous variable instead in the data analyses models used?

Was the sample size calculated powered to determine the factors associated with TB-related stigma?

17. Results, Discussion and Conclusion.

The main confusion for me is around denominators and the respective proportions related to TB stigma that have been presented. Clarification on this is needed.

18. Study limitations need to be acknowledged.

**********

6. PLOS authors have the option to publish the peer review history of their article (what does this mean?). If published, this will include your full peer review and any attached files.

**Do you want your identity to be public for this peer review?**   If you choose “no”, your identity will remain anonymous but your review may still be made public.

For information about this choice, including consent withdrawal, please see our Privacy Policy.

Reviewer #1: **Yes:**  Kampanart Chaychoowong

Reviewer #2: No

Reviewer #3: **Yes:**  Diana Marangu-Boore

**********

[NOTE: If reviewer comments were submitted as an attachment file, they will be attached to this email and accessible via the submission site. Please log into your account, locate the manuscript record, and check for the action link "View Attachments". If this link does not appear, there are no attachment files.]

-->Figure Resubmissions:

While revising your submission, we strongly recommend that you use PLOS’s NAAS tool (https://ngplosjournals.pagemajik.ai/artanalysis) to test your figure files. NAAS can convert your figure files to the TIFF file type and meet basic requirements (such as print size, resolution), or provide you with a report on issues that do not meet our requirements and that NAAS cannot fix.

After uploading your figures to PLOS’s NAAS tool - https://ngplosjournals.pagemajik.ai/artanalysis, NAAS will process the files provided and display the results in the "Uploaded Files" section of the page as the processing is complete. If the uploaded figures meet our requirements (or NAAS is able to fix the files to meet our requirements), the figure will be marked as "fixed" above. If NAAS is unable to fix the files, a red "failed" label will appear above. When NAAS has confirmed that the figure files meet our requirements, please download the file via the download option, and include these NAAS processed figure files when submitting your revised manuscript.

Attachment Submitted filename: Manuscript.pdf

Attachment Submitted filename: Review Report D Kimuli.docx

# Author comment: Author response to Decision Letter 1

**DOI:** 10.1371/journal.pgph.0005283.r002

2 Mar 2026

Attachment Submitted filename: Response Tracker_Final version.docx

# Peer review: Decision Letter 1

**DOI:** 10.1371/journal.pgph.0005283.r003

**Authors:** Graeme Hoddinott

25 Mar 2026

PGPH-D-25-02705R1

Settings, Characteristics, and Experiences of Stigma Among People with Tuberculosis in Kenya: National Survey Results

PLOS Global Public Health

Dear Dr. Ronoh,

Thank you for submitting your manuscript to PLOS Global Public Health. After careful consideration, we feel that it has merit but does not fully meet PLOS Global Public Health’s publication criteria as it currently stands. Therefore, we invite you to submit a revised version of the manuscript that addresses the points raised during the review process.

==============================

EDITOR:

Reviewers have raised a few more minor comments. Please address these and then we should be able to proceed to acceptance.

==============================

Please submit your revised manuscript by Apr 24 2026 11:59PM. If you will need more time than this to complete your revisions, please reply to this message or contact the journal office at globalpubhealth@plos.org. When you're ready to submit your revision, log on to https://www.editorialmanager.com/pgph/ and select the 'Submissions Needing Revision' folder to locate your manuscript file.

Please include the following items when submitting your revised manuscript:

- A letter that responds to each point raised by the editor and reviewer(s). You should upload this letter as a separate file labeled 'Response to Reviewers'.
- A marked-up copy of your manuscript that highlights changes made to the original version. You should upload this as a separate file labeled 'Revised Manuscript with Track Changes'.
- An unmarked version of your revised paper without tracked changes. You should upload this as a separate file labeled 'Manuscript'.

Guidelines for resubmitting your figure files are available below the reviewer comments at the end of this letter.

As the corresponding author, your ORCID iD is verified in the submission system and will appear in the published article. PLOS supports the use of ORCID, and we encourage all coauthors to register for an ORCID iD and use it as well. Please encourage your coauthors to verify their ORCID iD within the submission system before final acceptance, as unverified ORCID iDs will not appear in the published article. *Only*   the individual author can complete the verification step; PLOS staff *cannot*   verify ORCID iDs on behalf of authors.

We look forward to receiving your revised manuscript.

Kind regards,

Graeme Hoddinott, Ph.D

Academic Editor

PLOS Global Public Health

Journal Requirements:

1. Please provide a detailed online Financial Disclosure statement. This is published with the article. It must therefore be completed in full sentences and contain the exact wording you wish to be published.

a) Please clarify all sources of financial support for your study. List the grants, grant numbers, and organizations that funded your study, including funding received from your institution. Please note that suppliers of material support, including research materials, should be recognized in the Acknowledgements section rather than in the Financial Disclosure.

b) State the initials, alongside each funding source, of each author to receive each grant. For example: “This work was supported by the National Institutes of Health (####### to AM; ###### to CJ) and the National Science Foundation (###### to AM).”

c) State what role the funders took in the study. If the funders had no role in your study, please state: “The funders had no role in study design, data collection and analysis, decision to publish, or preparation of the manuscript.”

For more information, please go to our submission guidelines:

https://journals.plos.org/globalpublichealth/s/submission-guidelines#loc-financial-disclosure-statement

2. Please ensure that the funders and grant numbers match between the Financial Disclosure field and the Funding Information tab in your submission form. Note that the funders must be provided in the same order in both places as well.

3. In the online submission form, you indicated that "The data will be made readily available with any analytic scripts as may be required".

All PLOS journals now require all data underlying the findings described in their manuscript to be freely available to other researchers, either

a) In a public repository,

b) Within the manuscript itself, or

d) Uploaded as supplementary information.

This policy applies to all data except where public deposition would breach compliance with the protocol approved by your research ethics board. If your data cannot be made publicly available for ethical or legal reasons (e.g., public availability would compromise patient privacy), please explain your reasons by return email and your exemption request will be escalated to the editor for approval. Your exemption request will be handled independently and will not hold up the peer review process, but will need to be resolved should your manuscript be accepted for publication. One of the Editorial team will then be in touch if there are any issues.

For further assistance, you may go to: http://journals.plos.org/globalpublichealth/s/data-availability

4. Please provide separate main figure files in .tif or .eps format only and ensure that all files are under our size limit of 10MB.

For more information about how to convert your figure files please see our guidelines: https://journals.plos.org/globalpublichealth/s/figures

5. We note that the ethics approval documents should not be published with the paper and are for internal information only. Please change the file type to 'Other' to ensure that they are not published.

6. If the reviewer comments include a recommendation to cite specific previously published works, please review and evaluate these publications to determine whether they are relevant and should be cited. There is no requirement to cite these works unless the editor has indicated otherwise.

7. Please review your reference list to ensure that it is complete and correct. If you have cited papers that have been retracted, please include the rationale for doing so in the manuscript text, or remove these references and replace them with relevant current references. Any changes to the reference list should be mentioned in the rebuttal letter that accompanies your revised manuscript. If you need to cite a retracted article, indicate the article’s retracted status in the References list and also include a citation and full reference for the retraction notice.

Additional Editor Comments (if provided):

[Note: HTML markup is below. Please do not edit.]

Reviewers' comments:

Reviewer's Responses to Questions

**Comments to the Author**

1. If the authors have adequately addressed your comments raised in a previous round of review and you feel that this manuscript is now acceptable for publication, you may indicate that here to bypass the “Comments to the Author” section, enter your conflict of interest statement in the “Confidential to Editor” section, and submit your "Accept" recommendation.

Reviewer #1: All comments have been addressed

Reviewer #3: (No Response)

**********

2. Does this manuscript meet PLOS Global Public Health’s publication criteria? Is the manuscript technically sound, and do the data support the conclusions? The manuscript must describe methodologically and ethically rigorous research with conclusions that are appropriately drawn based on the data presented.

Reviewer #1: Yes

Reviewer #3: Partly

**********

3. Has the statistical analysis been performed appropriately and rigorously?

Reviewer #1: Yes

Reviewer #3: Yes

**********

4. Have the authors made all data underlying the findings in their manuscript fully available (please refer to the Data Availability Statement at the start of the manuscript PDF file)?

The PLOS Data policy requires authors to make all data underlying the findings described in their manuscript fully available without restriction, with rare exception. The data should be provided as part of the manuscript or its supporting information, or deposited to a public repository. For example, in addition to summary statistics, the data points behind means, medians and variance measures should be available. If there are restrictions on publicly sharing data—e.g. participant privacy or use of data from a third party—those must be specified.

Reviewer #1: Yes

Reviewer #3: Yes

**********

5. Is the manuscript presented in an intelligible fashion and written in standard English?

PLOS Global Public Health does not copyedit accepted manuscripts, so the language in submitted articles must be clear, correct, and unambiguous. Any typographical or grammatical errors should be corrected at revision, so please note any specific errors here.

Reviewer #1: Yes

Reviewer #3: Yes

**********

6. Review Comments to the Author

Please use the space provided to explain your answers to the questions above. You may also include additional comments for the author, including concerns about dual publication, research ethics, or publication ethics. (Please upload your review as an attachment if it exceeds 20,000 characters)

Reviewer #1: (No Response)

Reviewer #3: A few comments have not been fully addressed. The main manuscript submitted has comments. Although alternative terms for the word 'Pagan' have been provided by one of the co-authors (SM), this has not been changed.

Lines 151-152 seem incomplete: "Within these sub-counties, a total of 180 health facilities offering TB treatment services were

identified as primary recruitment sites for participants undergoincronbach’s"

The actual data to support the methods indicated are not available in the results. "Exploratory factor analysis (EFA) was conducted to test the internal consistency and construct

validity of the stigma scale in the Kenyan context. Cronbach’s alpha was calculated to assess

internal reliability, with values ≥0.7 indicating acceptable consistency. The principal components

extraction method was used to identify underlying factors, with factor loadings ≥0.4 considered

acceptable."

**********

7. PLOS authors have the option to publish the peer review history of their article (what does this mean?). If published, this will include your full peer review and any attached files.

**Do you want your identity to be public for this peer review?**   If you choose “no”, your identity will remain anonymous but your review may still be made public.

For information about this choice, including consent withdrawal, please see our Privacy Policy.

Reviewer #1: **Yes:**  Kampanart Chaychoowong

Reviewer #3: No

**********

[NOTE: If reviewer comments were submitted as an attachment file, they will be attached to this email and accessible via the submission site. Please log into your account, locate the manuscript record, and check for the action link "View Attachments". If this link does not appear, there are no attachment files.]

Figure Resubmissions:

While revising your submission, we strongly recommend that you use PLOS’s NAAS tool (https://ngplosjournals.pagemajik.ai/artanalysis) to test your figure files. NAAS can convert your figure files to the TIFF file type and meet basic requirements (such as print size, resolution), or provide you with a report on issues that do not meet our requirements and that NAAS cannot fix.

After uploading your figures to PLOS’s NAAS tool - https://ngplosjournals.pagemajik.ai/artanalysis, NAAS will process the files provided and display the results in the "Uploaded Files" section of the page as the processing is complete. If the uploaded figures meet our requirements (or NAAS is able to fix the files to meet our requirements), the figure will be marked as "fixed" above. If NAAS is unable to fix the files, a red "failed" label will appear above. When NAAS has confirmed that the figure files meet our requirements, please download the file via the download option, and include these NAAS processed figure files when submitting your revised manuscript.

# Author comment: Author response to Decision Letter 2

**DOI:** 10.1371/journal.pgph.0005283.r004

21 Apr 2026

Attachment Submitted filename: Tracker_Response to Reviewers Comments -21_04_2026.docx

# Peer review: Decision Letter 2

**DOI:** 10.1371/journal.pgph.0005283.r005

**Authors:** Graeme Hoddinott

30 Apr 2026

Settings, Characteristics, and Experiences of Stigma Among People with Tuberculosis in Kenya: National Survey Results

PGPH-D-25-02705R2

Dear Mr. Ronoh,

We are pleased to inform you that your manuscript 'Settings, Characteristics, and Experiences of Stigma Among People with Tuberculosis in Kenya: National Survey Results' has been provisionally accepted for publication in PLOS Global Public Health.

Before your manuscript can be formally accepted you will need to complete some formatting changes, which you will receive in a follow up email. A member of our team will be in touch with a set of requests.

Please note that your manuscript will not be scheduled for publication until you have made the required changes, so a swift response is appreciated.

IMPORTANT: The editorial review process is now complete. PLOS will only permit corrections to spelling, formatting or significant scientific errors from this point onwards. Requests for major changes, or any which affect the scientific understanding of your work, will cause delays to the publication date of your manuscript.

If your institution or institutions have a press office, please notify them about your upcoming paper to help maximize its impact. If they'll be preparing press materials, please inform our press team as soon as possible -- no later than 48 hours after receiving the formal acceptance. Your manuscript will remain under strict press embargo until 2 pm Eastern Time on the date of publication. For more information, please contact globalpubhealth@plos.org.

Thank you again for supporting Open Access publishing; we are looking forward to publishing your work in PLOS Global Public Health.

Best regards,

Graeme Hoddinott, Ph.D

Academic Editor

PLOS Global Public Health

***********************************************************

Reviewer Comments (if any, and for reference):

Reviewer's Responses to Questions

**Comments to the Author**

1. If the authors have adequately addressed your comments raised in a previous round of review and you feel that this manuscript is now acceptable for publication, you may indicate that here to bypass the “Comments to the Author” section, enter your conflict of interest statement in the “Confidential to Editor” section, and submit your "Accept" recommendation.

Reviewer #3: All comments have been addressed

**********

2. Does this manuscript meet PLOS Global Public Health’s publication criteria? Is the manuscript technically sound, and do the data support the conclusions? The manuscript must describe methodologically and ethically rigorous research with conclusions that are appropriately drawn based on the data presented.

Reviewer #3: Yes

**********

3. Has the statistical analysis been performed appropriately and rigorously?

Reviewer #3: Yes

**********

4. Have the authors made all data underlying the findings in their manuscript fully available (please refer to the Data Availability Statement at the start of the manuscript PDF file)?

The PLOS Data policy requires authors to make all data underlying the findings described in their manuscript fully available without restriction, with rare exception. The data should be provided as part of the manuscript or its supporting information, or deposited to a public repository. For example, in addition to summary statistics, the data points behind means, medians and variance measures should be available. If there are restrictions on publicly sharing data—e.g. participant privacy or use of data from a third party—those must be specified.

Reviewer #3: Yes

**********

5. Is the manuscript presented in an intelligible fashion and written in standard English?

PLOS Global Public Health does not copyedit accepted manuscripts, so the language in submitted articles must be clear, correct, and unambiguous. Any typographical or grammatical errors should be corrected at revision, so please note any specific errors here.

Reviewer #3: Yes

**********

6. Review Comments to the Author

Please use the space provided to explain your answers to the questions above. You may also include additional comments for the author, including concerns about dual publication, research ethics, or publication ethics. (Please upload your review as an attachment if it exceeds 20,000 characters)

Reviewer #3: (No Response)

**********

7. PLOS authors have the option to publish the peer review history of their article (what does this mean?). If published, this will include your full peer review and any attached files.

**Do you want your identity to be public for this peer review?**   If you choose “no”, your identity will remain anonymous but your review may still be made public.

For information about this choice, including consent withdrawal, please see our Privacy Policy.

Reviewer #3: No

**********
